# Polysaccharide Hydrogel Combined with Mesenchymal Stem Cells Promotes the Healing of Corneal Alkali Burn in Rats

**DOI:** 10.1371/journal.pone.0119725

**Published:** 2015-03-19

**Authors:** Yifeng Ke, Yixiang Wu, Xuan Cui, Xun Liu, Min Yu, Chunbo Yang, Xiaorong Li

**Affiliations:** 1 TianjinMedical University Eye Hospital, The College of Optometry,Tianjin Medical University Eye Institute, Tianjin, China; 2 School of Public Health, Tianjin Medical University, Tianjin, China; Cedars Sinai Medical Center, UNITED STATES

## Abstract

Corneal chemical burns are common ophthalmic injuries that may result in permanent visual impairment. Although significant advances have been achieved on the treatment of such cases, the structural and functional restoration of a chemical burn-injured cornea remains challenging. The applications of polysaccharide hydrogel and subconjunctival injection of mesenchymal stem cells (MSCs) have been reported to promote the healing of corneal wounds. In this study, polysaccharide was extracted from Hardy Orchid and mesenchymal stem cells (MSCs) were derived from Sprague-Dawley rats. Supplementation of the polysaccharide significantly enhanced the migration rate of primarily cultured rat corneal epithelial cells. We examined the therapeutic effects of polysaccharide in conjunction with MSCs application on the healing of corneal alkali burns in rats. Compared with either treatment alone, the combination strategy resulted in significantly better recovery of corneal epithelium and reduction in inflammation, neovascularization and opacity of healed cornea. Polysaccharide and MSCs acted additively to increase the expression of anti-inflammatory cytokine (TGF-β), antiangiogenic cytokine (TSP-1) and decrease those promoting inflammation (TNF-α), chemotaxis (MIP-1α and MCP-1) and angiogenesis (VEGF and MMP-2). This study provided evidence that Hardy Orchid derived polysaccharide and MSCs are safe and effective treatments for corneal alkali burns and that their benefits are additive when used in combination. We concluded that combination therapy with polysaccharide and MSCs is a promising clinical treatment for corneal alkali burns and may be applicable for other types of corneal disorder.

## Introduction

Corneal chemical burns are common ophthalmic emergencies accounting for 7.7–18% of all ocular traumas, with alkali injuries being more common than those from acid [1]. In severe cases, the eye limbus and central epithelium can be lost leading to loss of vision [2]. Slow epithelialization, persistent ulceration, corneal perforation and angiogenesis are the main complications and result from the processes of inflammation, neovascularization, and conjunctivalization of cornea [3–5].

Strategies to treat corneal chemical burns include antibiotics, tear substitutes, corticosteroids, ascorbic acid, collagenase inhibitors and surgical treatments such as penetrating keratoplasty and amniotic membrane transplantation [6]. However, the structural and functional restoration of alkali burn-injuries to the cornea remains a challenge, despite these therapies and thus prompting the search for novel treatment strategies including the use of polysaccharides and stem cell based therapies.

Polysaccharides are high molecular weight carbohydrates composed of long chains of monosaccharide units bound together by glycosidic bonds. At least five different types of polysaccharide exist in corneal stroma, i.e. keratin sulphate, chondroitin-4-sulfate chondroitin, dermatan sulfate and heparan sulfate which play important roles in maintaining the water content and transparency of the cornea [7]. It was reported that the levels of endogenous polysaccharides were reduced in the healing area of perforating corneal [8].

Treatment with sodium hyaluronate promoted corneal wound healing through the stimulation of epithelial cell proliferation, the promotion of epithelial cell adhesion to stroma fibronectin and the suppression of inflammatory cell infiltration [9–11]. Polysaccharides may also be beneficial in the treatment of corneal diseases involving neovascularization and inflammation [12,13]. The antinociceptive and anti-inflammatory activities of polysaccharides and their impacts on cellular proliferation and the immune response have been widely reported as well [14, 15].

Hardy Orchid has been shown to facilitate the treatment of canal mucosal damage, ulcers, bleeding, bruises and burns. Luo et al. found that Hardy Orchid polysaccharide controlled the inflammatory responses and accelerated the wound closure in a mouse model of cutaneous trauma [16]. Diao et al. reported that Hardy Orchid polysaccharide modulated the expression of pro-inflammatory cytokines including NO, TNF-α, and interleukin 1 beta (IL-1β) in a murine macrophage-like cell line [17].

Mesenchymal stem cells (MSC) are multipotent stromal cells derived from the mesoderm that can differentiate into a variety of cell types. MSCs are relatively easy to isolate and expand making them promising candidates for cell replacement therapy. Recently, MSCs have been studied for the treatment of corneal chemical burn with encouraging results [18,19]. Treatment with subconjunctival MSCs has been found to significantly enhance the recovery of the corneal epithelium and decrease the area of corneal neovascularization [20]. Transplanted MSCs are thought to promote corneal wound healing through secretion of anti-inflammatory and anti-angiogenic cytokines.

In the current study, we explored the effect of topical polysaccharide therapy in combination with subconjunctival administration of MSCs on the healing of corneal alkali burns.

## Materials and Methods

### Extraction, purification and characterization of polysaccharides

The plant Hardy Orchid was grounded and dispersed in 80°C distilled water for 4h and filtered to remove impurities. The filtrate was treated with 3x vol. of 95% ethanol overnight, and the precipitate was washed with 95% ethanol and resolved in distilled water. To exclude protein components, 1/3 vol. of chloroform/n-butanol (4:1 v/v) was added into the water solution and mixed by vigorous shaking. After chloroform water phase separation the upper aqueous phase was collected. The above procedure was repeated three times. The final aqueous phase was dialyzed at a cut-off of 3000–5000 Da and the concentrated solution was treated with 3x vol. of 95% ethanol overnight. The precipitate was lyophilized to give a crude extract. The crude extract was dissolved and applied onto Sephadex G-100 column for further purification and lyophilized to obtain polysaccharide powder.

To analyze glucosidic bonds and functional groups of the polysaccharide, 2 mg of the polysaccharide powder was mixed with KBr to obtain KBr pellet and subjected to infrared (IR) spectrum analysis using Nicolet 6700 FT-IR Spectrometer (PT.Contitus.Co.LTD) with a scan range of 4000–500 cm^-1^.

Monosaccharides composition of the polysaccharide was analyzed by PMP derivation high performance liquid chromatography (HPLC). Briefly, 2mg polysaccharide powder was dissolved in 2 M trifluoroacetic acid (TFA) and completely hydrolyzed to monosaccharide at 105°C for 4 h. The solution was derived with 1-Phenyl-3-methyl-5-pyrazolone (PMP) and NaOH and subjected to analysis. 2mg of individual standard monosaccharides including D-mannose (Man), L-rhamnose (Rha), D-glucuronic acid (GlcUA), D-galacturonic acid (GalUA), D-glucose (Glc), D-galactose (Gal), D-xylose (Xyl), L-arabinose (Ara), Fucose (Fuc) were derived in the same way and subjected to analysis.

The purified polysaccharide was thiolated by esterification with thioglycolic acid in the presence of hydrochloric acid. Polysaccharide was dissolved in distilled water at 100mg/ml and 3.6ml of thioglycolic acid and 2ml of 7N HCl was added to allow reaction at 80°C for 180 min. Thiolated polysaccharide was precipitated using methanol and lyophilized. The thiolated polysaccharide was dissolved in PBS at 20mg/ml.

To generate polysaccharide hydrogel, we prepared maleimidized polylysine as crosslinker. In brief, polylysine solution (20mg/ml) was mixed with succinimide sulfate solution (5mg/ml) with volume ratio of 1:10 and continually stirred at room temperature for 2 hours. After dialysis and lyophilisation, maleimidized polylysine was obtained and dissolved in PBS at 20mg/ml. When thiolated polysaccharide was mixed with maleimidized polylysine at pH 7, the sulfhydryl groups reacted with maleimide to form stable thioether bonds to trigger the formation of polysaccharide hydrogel.

### Isolation and culture of bone marrow mesenchymal stem cells (BMSCs)

Bone marrow mesenchymal stem cells (BMSCs) were derived from six-week-old female Sprague-Dawley rats weighing 180–220 g (Tianjin Medical University animal Center for Experiment, Tianjin, China). All rats were sacrificed with an overdose of 10% chloral hydrate. In brief, bone marrow was harvested from the long bones and layered onto Lymphoprep (1.077 g/mL; Nycomed, Birmingam, UK) for centrifugation at 850 g, 25 min. The isolated mononuclear cells were cultured in Dulbecco’s modified Eagle medium (DMEM) containing10% fetal bovine serum (FBS), 100 U/mL penicillin, 100 mg/mL streptomycin, 2.5 ug/mL fungizone and 2 mM L-glutamine (Invitrogen, Paisley, UK) and incubated at 37°C in a humidified atmosphere with 5% CO2. The BMSCs at passages 3 were used for all experiments described.

### Animals

Six-week-old female Sprague-Dawley weighing 180–220 g were used (Tianjin Medical University animal center, Tianjin, China). At the end of the experiment, all rats were sacrificed with an overdose of 10% chloral hydrate. Only the right eye of each rat was used. All procedures used in this study were in accordance with the principles of the ARVO Statement for the Use of Animals in Ophthalmic and Vision Research. The study was approved by the Institutional Animal Care and Use Committee of Tianjin Medical University Eye Hospital.

### Rat corneal epithelial cells (rCECs) culture and identification

The corneas of the rats were harvested and washed twice with Hanks balanced salt solution buffer (Sigma Chemicals). After careful removal of excessive corneal stroma, the tissue was cut into small pieces, placed onto culture plate and cultured with medium containing Dulbecco’s modified Eagle medium (DMEM), 5 ng/ml of epidermal growth factor (EGF), 1% glutamax, 1% non-essential amino acids, 1% antibiotic solution and 5% FBS. The cultured CECs were identified by CK-3 immunofluorescence and the percentage of CK3-positive cells was examined by flow cytometry. CK3 antibody was from Biobyt (orb5866).

### MTT cell proliferation assay

The proliferation of rat corneal epithelial cells (rCECs) was examined using 3-(4, 5)-dimethylthiahiazo (-z-y1)-3, 5-di-phenytetrazoliumromide (MTT) labeling assay. rCECs were incubated in a medium with 0, 50, 100, 200 μg/ml of Polysaccharide for 72 h followed by 4 h incubation with MTT. The MTT transformed crystals were dissolved in dimethyl sulfoxide, and absorbance at 490 nm was measured using a microplate reader (infinite M200PRO, Tecan, Switzerland).

### Wound healing assay

The effect of polysaccharide on the migration of rat corneal epithelial cells (rCECs) was evaluated using a wound healing assay. Briefly, the cells were plated on 24 well culture plates coated with 1% gelatin. A scratch was made with a micropipette tip after confluence was reached. Cultures were then rinsed to remove detached cells and cultured with medium containing various concentrations of polysaccharide or with the solvent control. The cell migration rates were calculated 24h later with the formula m = (1-n/r)×100%, where n is the width of scratch at 24h, r is the initial width of scratch.

### Animal model of corneal alkali burn and treatments

A corneal alkali burn was generated in the right eye of each rat (200 rats). The rats were anesthetized by an intramuscular injection of 40 mg/kg ketamine and 10 mg/kg xylazine hydrochloride. A piece of filter paper (6-mm diameter) soaked with 4 ul NaOH (1 mol/l) was applied to the center of the cornea for 30 seconds. The cornea was then rinsed with 30 ml of saline for 30 seconds.

After the corneal alkali burn, animals were randomly divided into four groups: control group (n = 50), MSC group (n = 50), polysaccharide group (n = 50) and PM group (polysaccharide hydrogel combined with MSCs application, n = 50). Immediately and 2 days after the corneal alkali burn, various treatments were carried out twice separately. Animals in the MSC group received subconjunctival injections of 2x10^6^ MSCs in 0.1 ml PBS. In the polysaccharide group, 50ul polysaccharide solution (200ug/ml) was applied onto the surface of the injured cornea and polylysine crosslinker solution was dropped on top subsequently to form hydrogel within 30 seconds. In the PM group, polysaccharide hydrogel was applied in conjunction with subconjunctival injection of MSCs. The control group was treated with subconjunctival injection and topical application of PBS. Levofloxacin was applied to the experimental eyes twice a day throughout the experiment.

Animal condition was monitored three times every day during the first 7 days post-injury. Thereafter animal condition was monitored once a day until the end of the experiment. On day 3 and 14 post-injury, 5 rats from each group were sacrificed and cornea excised for histological examination; on day 3, 7, 14 and 28 post-injury, RNAs were extracted from 5 corneas in each group for qRT-PCR examination; on day 3, 7 and 14 post-injury, total proteins were extracted from 5 corneas in each group for ELISA examination; on day 28 postinjury, ink perfusion via the aorta was performed with 5 rats in each group and cornea excised for observation of neovascularization.

### Observation and examination

General observations were performed including corneal epithelial recovering, corneal clarity, and corneal neovascularization. All observations were performed by a single experienced ophthalmologist who was blind to the allocation of the animals in each group. The corneal epithelial integrity was observed by corneal fluorescence staining at day 3 and day 7 after the corneal alkali burn. Corneal opacity was examined by slit-lamp (SL-120; Zeiss, Jena, Germany) at 3, 7 and 14 days after the corneal alkali burn and graded as described by Sonoda and Streilein [21]: grade 0, completely transparent cornea; grade 1, minimal corneal opacity, but iris clearly visible; grade 2, mild corneal opacity, iris vessels still visible; grade 3, moderate corneal opacity, pupil margin but not iris vessels visible; grade 4, complete corneal opacity, pupil not visible. Corneal neovascularization was quantified by calculating the wedge-shaped area of vessel growth using the following equation [22]: A = C / 12 × 3.1416 [r^2^ × (r − l)^2^], where A is the area, C is a fraction of circumference based on 12-h clock, l is the radius from the center to the border of vessel growth, and r is the radius of the cornea. The amount of neovascularization was compared between the groups using the ratio of the neovascularized area to the whole corneal area. An image analyzer (Image Pro Plus 6.0; Media Cybernetics) was used for measuring the radius. To evaluate the development of corneal neovascularization (CNV), ink perfusion via the aorta was performed on day 28 as described previously [23]. Thereafter, the eyes were fixed in 10% neutralized buffered formaldehyde overnight, and the corneas were dissected and flattened for imaging.

### Hematoxylin and eosin staining histology

Eyeballs were fixed with formalin and paraffin-embedded. Serial sections (3μm) were deparaffinized by sequential washing with xylene followed by washing with descending series of ethanol and then processed for hematoxylin and eosin staining. The stained sections were observed under microscope (Olympus, Tokyo, Japan).

### Quantitative Real-Time PCR (qRT-PCR)

Total RNAs were extracted from 5 corneas in each group with TRIzol (Invitrogen). 100 ng RNA was reverse transcribed into cDNA via Oligo (dT) with PrimeScript RT reagent kit (Takara). Real-time quantitative PCR was performed on a 7900HT Fast Real-Time PCR System (Applied Biosystems). All experiments were carried out in triplicate with SYBR Green JumpStart Taq ReadyMix (Sigma). The sequences of the PCR primers for TNF-α, TGF-β, TSP-1, MIP-1α, MCP-1, MMP-2 and VEGF are listed in S1 Table.

### Enzyme-linked immunosorbent assay (ELISA)

Corneas excised from the eyeballs were sliced and lysed with tissue extraction reagent (life technologies FNN0071). The total protein concentrations were measured using BCA protein assay kit (thermo scientific 23235). 20 μg total protein from each sample was applied for measurement of VEGF and TGF-β using ELISA kits (R&D Systems, Minneapolis, MN) at 490 nm. ELISA experiments were performed in triplicate.

### Statistical analysis

Analysis of variance (single factor or two factors ANOVA with replication) was used to compare multiple samples (at one time or several time points).P value <0.05 was considered statistically significant (SPSS 12.0; SPSS, Chicago, IL).

## Results

### Hardy Orchid-derived polysaccharide showed compatibility with corneal epithelial cells and promoted cell migration

**Fig 1:**
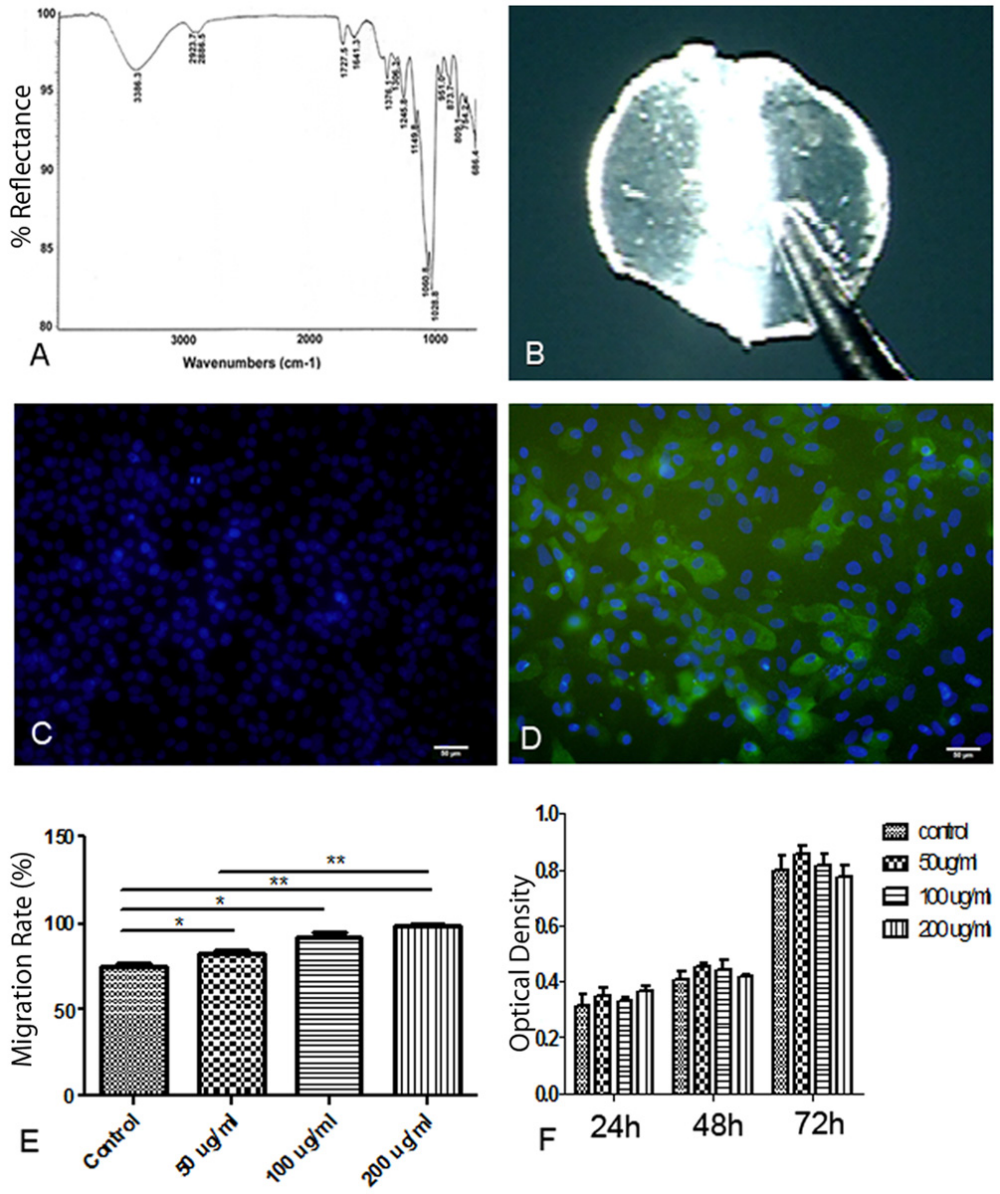
Characterization of Hardy Orchid-derived polysaccharide and examination of its impacts on migration and proliferation of rat corneal epithelial cells (rCECs). (A) Infrared spectrum analysis of Hardy Orchid-derived polysaccharide, (B) In vitro formed polysaccharide hydrogel composite, (C) Negative control for Cytokeratin-3 immunostaining of rCECs cultured with polysaccharide supplementation, (D) Cytokeratin-3 immunostaining of rCECs cultured with polysaccharide supplementation, (E) The migration rate of rCECs in control and polysaccharide treated groups 24h after wound. Polysaccharide treatment increased rCECs migration rate in a dose-dependent manner, (F) Proliferation analysis of rCECs in control and polysaccharide treated groups. At all three time points there were no significant differences between control and various polysaccharide dose groups. Data are presented as mean ± standard deviation (n = 5). One-way (E) or two-way (F) ANOVA analysis was performed to determine the significance of the difference between various treatment groups (* p<0.05, **p<0.01, ***p<0.001).

To analyze glucosidic bonds and functional groups of the polysaccharide, infrared (IR) spectrum analysis was carried out (Fig. 1A). According to the IR spectrum, Hardy Orchid—derived polysaccharide had typical polysaccharide absorption profile. The wide peak at 3600–3200 cm^-1^ indicated O-H stretching vibration, the peak at 3000–2750 cm^-1^ was C-H stretching vibration. The 1800–700 cm^-1^ region characterized the types of glucoside, substituent and epimer. Peaks in the 1376–1028 cm^-1^ band were characteristic hydrocarbon keys of pyranose. The characteristic absorption at 951–875 cm^-1^ suggested that the polysaccharide contains β-glucosyl residues and 809 cm^-1^ peak revealed the existence of mannose. Lack of absorption at 840 cm^-1^ and presence of absorption at 873 cm^-1^ indicated that monosaccharide residues were subject to β-glycosidic bond connection rather than α-type.

Monosaccharides composition of the polysaccharide was analyzed by PMP derivation high performance liquid chromatography (HPLC). By comparison of HPLC spectrum profile of polysaccharide with that of standard monosaccharides it was found that Hardy Orchid derived polysaccharide was mainly composed of D-mannose (Man) and D-glucose (Glc) with relative mole ratio of 6:1 according to their corresponding peak areas (S2 Fig.). Using BCA protein assay (thermo scientific 23235), a small amount of protein component was determined (0.04mg/ml) in the polysaccharide extract.

When mixed with polylysine crosslinker solution 1:1 at room temperature, the polysaccharide solution gelled within 30 seconds as a semi-transparent construct (Fig. 1B). We took advantage of this phenomenon to develop a polysaccharide treatment strategy in which this stable hydrogel was formed on the corneal surface, thereby ensuring intimate contact between the polysaccharide and the corneal epithelium.

To examine the compatibility and cytotoxicity of the polysaccharide with rat corneal epithelial cells (rCECs), polysaccharide solution was added into the culture of rCECs at concentrations of 50–200 μg/ml, after which their morphology, proliferation and migration characteristics were assessed.

In polysaccharide-supplemented culture, rCECs showed normal morphology identical to control rCECs cultured without polysaccharide supplementation. Immunostaining of cytokeratin-3 confirmed the corneal epithelial identity of the culture (Fig. 1C, D). There were no significant differences in the results of MTT assays at 24, 48 and 72 hours of culture between the polysaccharide and control groups, indicating that the polysaccharide treatment did not affect the proliferating rate of rCECs (Fig. 1E). A wound healing assay was performed to observe the effect of polysaccharide on rCECs migration. Compared to the control group, the 24 hour migration rates of rCECs were significantly increased in a dose-dependent manner when treated with polysaccharide, indicating that polysaccharide supplementation enhanced rCECs migration (Fig. 1F).

### Polysaccharide hydrogel and mesenchymal stem cells promoted the recovery of corneal epithelium and enhanced corneal clarity

After the establishment of corneal alkali burn model, the animals were divided into four groups. The polysaccharide group was treated with topical application of polysaccharide hydrogel. The MSC group received subconjunctival injection of mesenchymal stem cells suspended in PBS. The PM group had both treatments simultaneously. The control group was treated with topical application and subconjunctival injection of PBS.

**Fig 2:**
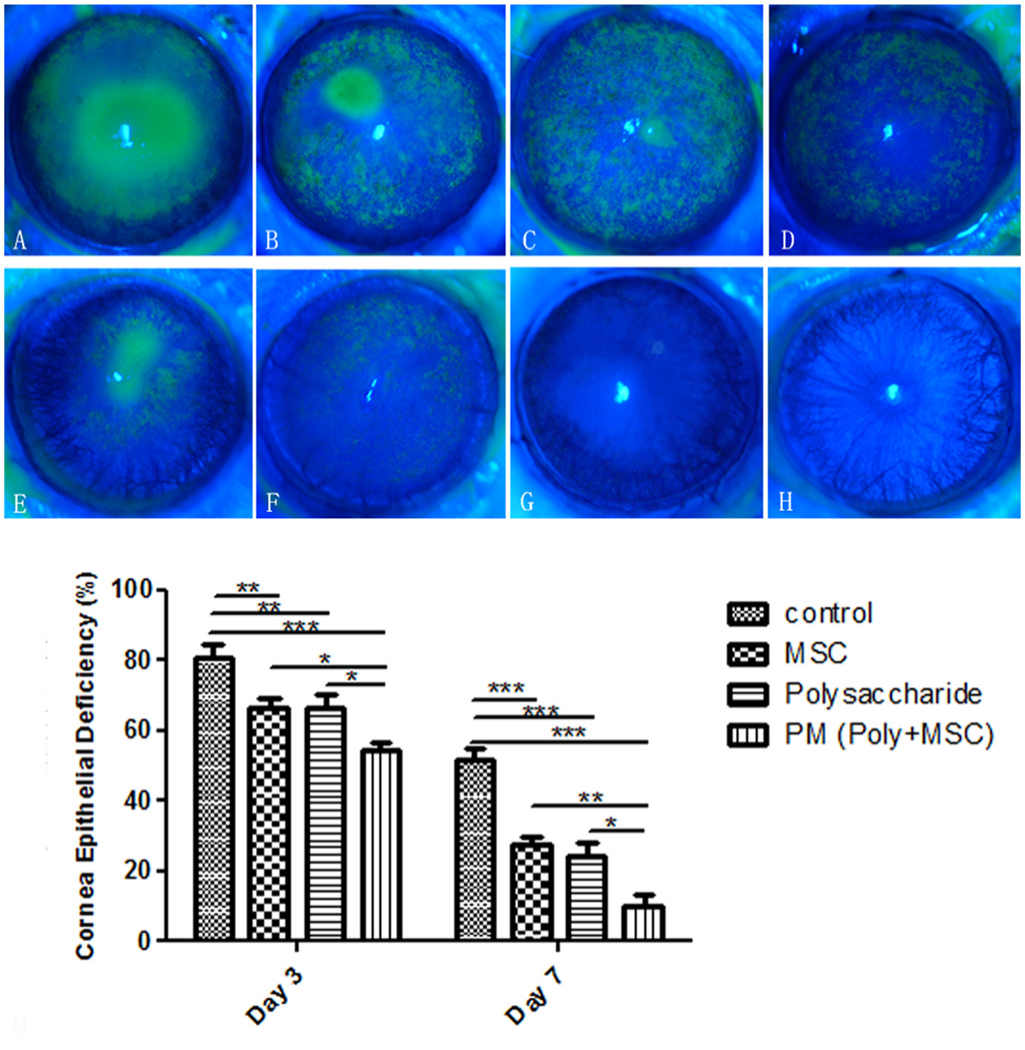
Re-epithelialization examination of alkali burn injured cornea. (A-D) Fluorescence staining of corneal epithelium 3 days after injury in control, MSCs treatment, polysaccharide treatment and polysaccharide-MSCs combination (PM) groups, (E-H) Fluorescence staining of corneal epithelium 7 days after injury in various groups, (I) statistics of corneal defect area ratios in various groups on dya3 and day7 post-injury. Data are presented as mean ± standard deviation (n = 5). Two-way ANOVA analysis was performed to determine the significance of the difference between various treatment groups (* p<0.05, **p<0.01, ***p<0.001). At both time points, MSCs and polysaccharide treatments significantly enhanced the recovery of corneal epithelium. Polysaccharide-MSCs combination (PM) groups showed additive effects compared with single treatment groups.

To determine the effects of the polysaccharide and/or MSCs on the recovery of corneal epithelium, fluorescein staining was used to measure the epithelial defect areas in various treatment groups at 3 and 7 days after alkali burn. On day 3 post-injury, the corneal defect area in the control group accounted for 80% of the corneal surface, which was significantly decreased to around 65% in both polysaccharide and MSCs treatment groups. When combined, polysaccharide and MSCs treatments showed an additive effect, resulting in a defect area of around 50% of the corneal surface. On day 7, the corneal defect area in all the groups was notably reduced. In control group, a defect area of around 50% remained. The polysaccharide and MSCs groups showed a much better appearance than the control group with only 20–30% defect area left. Notably, in the polysaccharide plus MSCs group, defect area decreased to 10% and the wound re-epithelialised completely in some individuals (Fig. 2).

**Fig 3:**
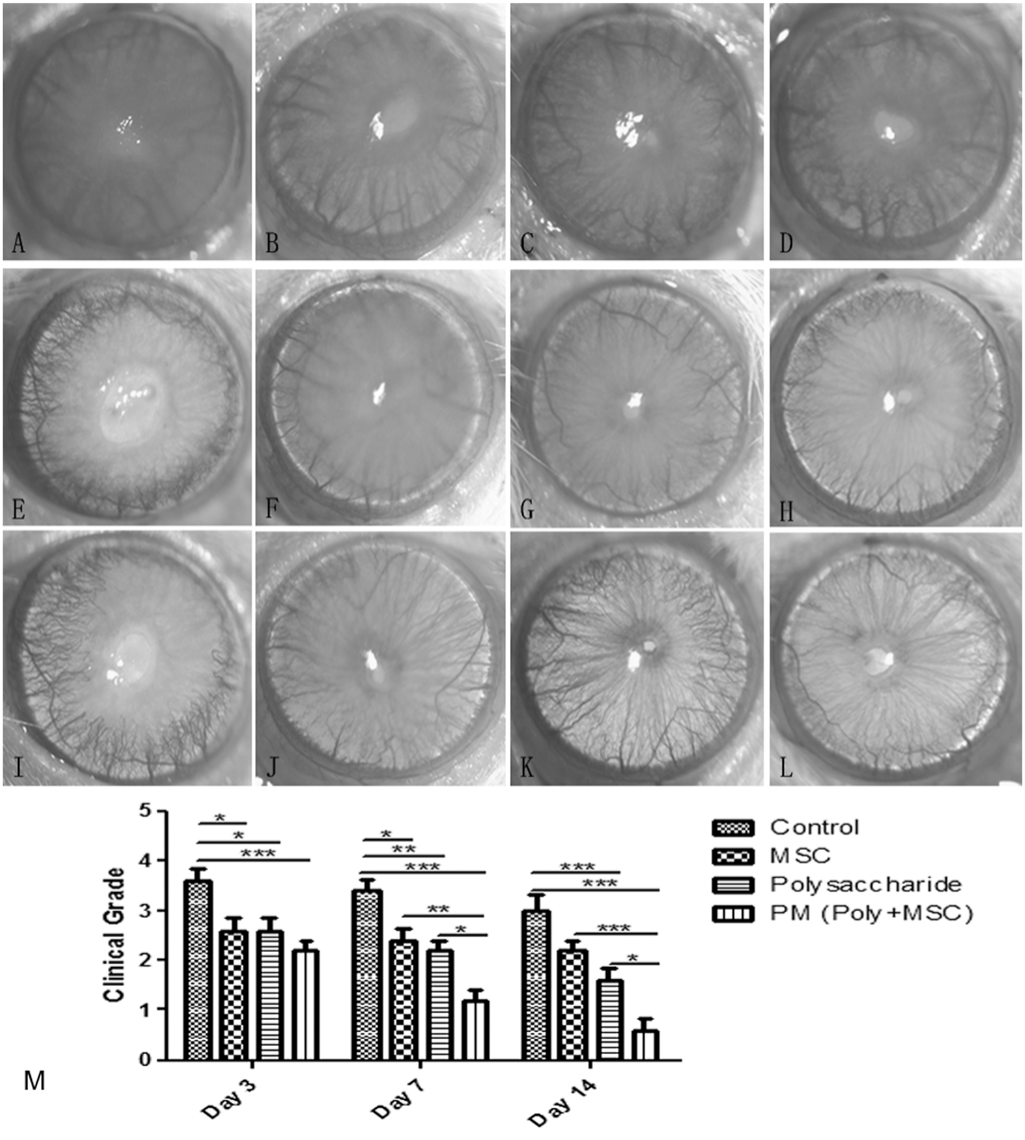
Corneal opacity evaluation of alkali burn injured corneas. (A-D) Corneal surface observation 3 days after injury in control, MSCs treatment, polysaccharide treatment and polysaccharide-MSCs combination (PM) groups, (E-H) Corneal surface observation 7 days after injury in various groups, (I-L) Corneal surface observation 14 days after injury in various groups, (M) statistics of corneal opacity grades in various groups on day3,7,14 post-injury. Data are presented as mean ± standard deviation (n = 5). Two-way ANOVA analysis was performed to determine the significance of the difference between various treatment groups (* p<0.05, **p<0.01, ***p<0.001). MSCs and polysaccharide treatments significantly decreased corneal opacity and Polysaccharide-MSCs combination (PM) groups showed additive effects compared with single treatment groups.

Corneal transparency in each group was evaluated using the slitlamp photography based on the principles described by Sonoda and Streilein [21]. From day 3 through day 14 post-injury, the control group showed severe corneal opacity graded as 3–3.6. Compared to the control group, polysaccharide and MSCs applications significantly reduced the grade of corneal opacification. Of note, after polysaccharide treatment, the corneal opacity grade decreased from 2.5 on day 3 to 1.5 on day 14. Furthermore, the polysaccharide and MSCs combination further improved corneal transparency, resulting in minimal opacity, graded at 2.2, 1.2 and 0.8 on day 3, day 7 and day 14 post-injury separately. Compared to single treatment groups, polysaccharide and MSCs combination showed significant additive effect on day 7 and day 14 post-injury (Fig. 3).

### Polysaccharide hydrogel and mesenchymal stem cells suppressed corneal neovascularization

Neovascularization is one of the major complications of chemical corneal burns and is closely related to prognosis. The development of corneal neovascularization (CNV) in various groups was quantified by calculating the wedge-shaped area of vessel growth.

**Fig 4:**
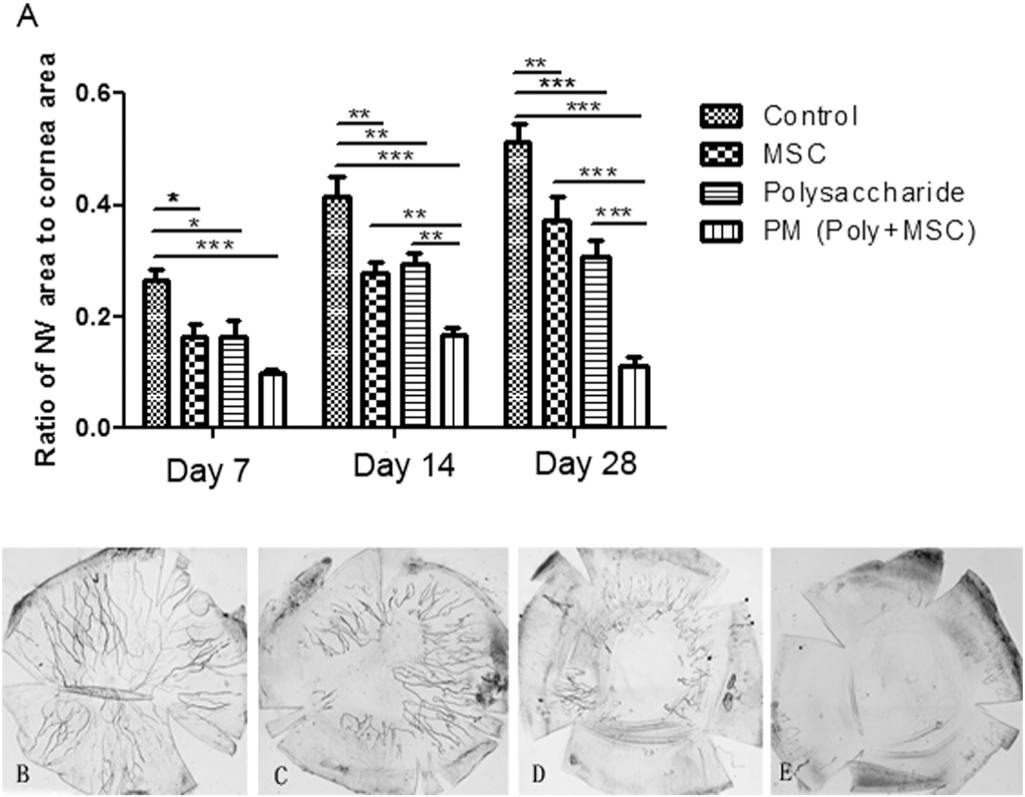
Analysis of corneal neovascularization after corneal alkali burn. (A) Ratio of neovascularization area to corneal area in control, MSC, polysaccharide and PM groups at day7, 14, 28 days post-injury, (B-E) Ink perfusion detection of corneal neovascularization on day 28 post-injury in control, MSC, and PM groups. Statistics data are presented as mean ± standard deviation (n = 5). Two-way ANOVA analysis was performed to determine the significance of the difference between various treatment groups (* p<0.05, **p<0.01, ***p<0.001). MSCs and polysaccharide treatments significantly decreased corneal neovascularization and Polysaccharide-MSCs combination (PM) groups showed additive effects compared with single treatment groups.

From day 7 to day 28 post-injury, the control group showed gradually increased area of CNV, which is consistent with the pathological process of corneal chemical burn. In contrast, the areas of CNV in polysaccharide and MSCs treatment groups were significantly less than that in the control group at all three time points. On day 28 post-injury, the ratio of CNV area to cornea area was 0.51 in the control group, which was 0.37 and 0.30 in the MSC and polysaccharide group respectively. Of note, from day 7 through day 28 post-injury, the area of CNV remained at low level in the combined therapy group in contrast to the gradual increase observed over this time in other groups. On day 7, the CNV ratio was 0.10 in the PM group, which increased a bit to 0.17 on day 14 then decreased to 0.11 on day 28. Our study revealed that when used in isolation, polysaccharide and MSC therapy reduced the area of neovascularization to a similar degree and that their effects were additive when used in combination. In addition, the sustained effect of combination therapy on neovascularization was highlighted (Fig. 4A).

To further confirm the results by slit lamp and image analyzing, ink perfusion via the aorta was performed on day 28 post-injury to identify corneal neovascularization. The control group showed remarkable neovascularization and the new vessels extended into the central area of the cornea. MSCs and polysaccharide treatment groups showed significantly less new vessels compared to the control group, consistent with the CNV area calculation results. Notably, PM group showed the least neovascularization confirming the additive suppression effect of MSCs and polysaccharide especially at late stages of corneal healing post-injury (Fig. 4B-E).

### Histological examination

**Fig 5:**
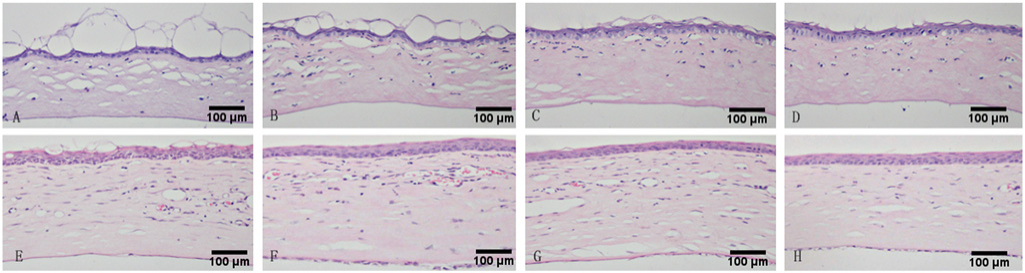
Histological examination of cornea in the control, MSCs, polysaccharide and PM groups at day3 (A-D), and day14 post injury (E-H).

Histological examination was performed on day 3 and day 14 after injury. On day 3, serious defects with bullous keratopathy in the corneal epithelium were identified in the control group. The defects were milder in the groups treated with either MSCs or polysaccharide and no bullous keratopathy was detected in the combination treatment group (Fig. 5).

On day 14 post-injury, histological examination of the control group revealed multiple layers of irregularly aligned, regenerating corneal epithelial cells, persistent bullous keratopathy, extensive inflammatory infiltrates and neovascularization within the stoma. In the polysaccharide and MSC groups the regenerating epithelial cells were more regularly aligned and there were fewer inflammatory infiltrates and less neovascularization in the stroma. In the PM group, no abnormalities were detected in the corneal epithelium. Few inflammatory cells were detected and little neovascularization was observed in the stroma. In addition, the corneal thickness was least in the PM suggesting the suppression of oedema (Fig. 5).

Thus, histological examination provided further evidence of the therapeutic effects of polysaccharide and MSC application on alkali burn-injured cornea.

### Examination of cytokines related to inflammation and neovascularization

**Fig 6:**
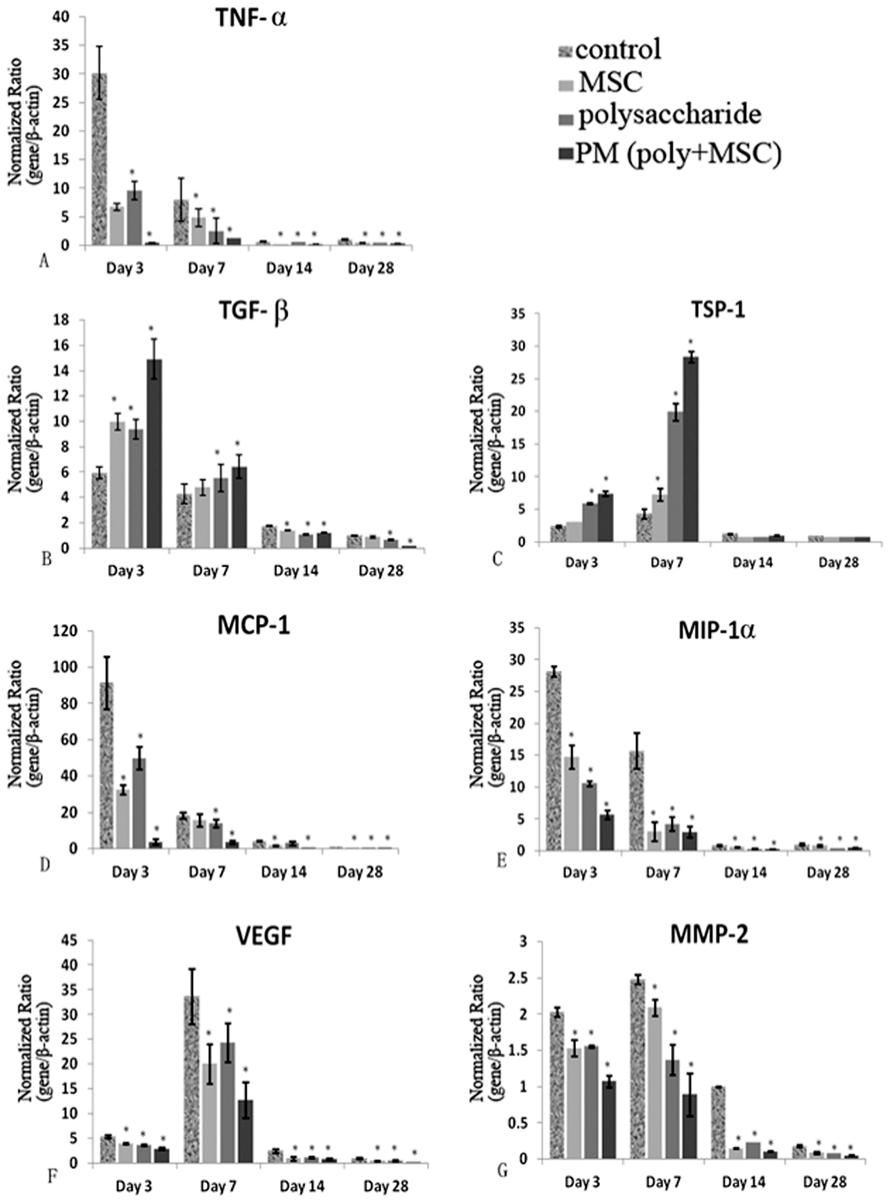
Quantitative Real-time PCR examination of inflammation, chemotaxis and neovascularization related genes. The data are presented as mean ± SD from three assays. Two-way ANOVA analysis was performed to determine the significance of the difference between various treatment groups (* p<0.05, **p<0.01).

To explore the mechanisms by which the polysaccharide and MSC therapies promoted the healing of corneal alkali burns, the expression level of cytokines related to inflammation, chemotaxis and neovascularization were examined by real-time PCR. On day 3, 7, 14 and 28 days post-injury, expression of the genes encoding the immunostimulatory cytokine TNF-α and the chemotactic factors MIP- 1α and MCP-1 were significantly lower in the polysaccharide and MSCs groups than in the control group. PM group showed the lowest expression at all the time points. Of note, the greatest reduction in expression of these genes was seen in the PM group on day 3 post-injury. On the contrary, expression of the gene for the anti-inflammatory cytokine TGF-β was significantly increased by treatment of polysaccharide and MSCs and the same additive effect was observed when used in combination. VEGF and MMP-2 stimulate angiogenesis following corneal chemical-burns. Expression of the VEGF gene increased in all groups up until day 7 and remarkably decreased thereafter. The expression of the genes encoding both VEGF and MMP-2 was significantly lower in the single treatment groups and was lowest in the PM group compared to the control group. In contrast, expression of gene for the antiangiogenic cytokine showed an opposite dynamics (Fig. 6).

**Fig 7:**
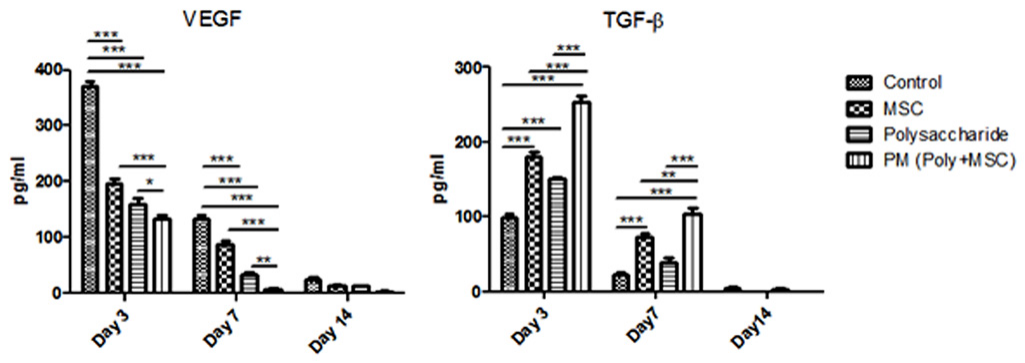
Evaluation of expression level of cytokines VEGF and TGF-β by ELISA. The data are presented as mean ± SD from three assays. Two-way ANOVA analysis was performed to determine the significance of the difference between various treatment groups (* p<0.05, **p<0.01, ***p<0.001).

To further confirm the effects of polysaccharide and MSCs on the expression level of cytokines, ELISA assay was performed to detect the cellular protein content of VEGF and TGF-β. The changes in TGF-β and VEGF genes expression were well mirrored by the results of ELISA on day 3, 7, and 14 post-injury (Fig. 7). At each time point, polysaccharide and MSCs groups showed decreased level of VEGF compared to the control group, and the PM group had the lowest. In contrast to VEGF, TGF-β showed the opposite dynamic scenario, which was increased by treatment of polysaccharide/MSCs alone or in combination additively.

## Discussion

In recent years, polysaccharide has been widely reported for the experimental therapies of corneal disorders such as corneal incisions [24], Corneal epithelial abrasion and alkali burn injuries [25], dry eye syndrome [26] etc. In addition, numerous reports have described therapeutic benefits of MSCs administration on corneal wound healing [18,19].

In the current study, polysaccharide hydrogel derived from Hardy Orchid and mesenchymal stem cells were applied for the treatment of corneal alkali burn. Compared with control group, single treatment of polysaccharide hydrogel or MSCs significantly promoted corneal epithelium recovery, suppressed inflammation, neovascularization, and greatly alleviated the haze and opacity of injured cornea. When polysaccharide hydrogel and MSCs were applied concurrently, additive effect was observed and the cornea showed the highest recovering rate of epithelium, best clarity, least inflammatory infiltration and mildest neovascularization. To our knowledge, this is the first report that combination of polysaccharide hydrogel and MSCs additively promoted the healing of corneal alkali burn.

Hardy Orchid has the functions of hemostasia, detumescence and recovery enhancement, therefore has been widely used to treat alimentary canal mucosal damage, ulcers, bleeding, bruises and burns [17]. Our study provides evidence of biocompatibility and safety of Hardy Orchid polysaccharide treatment for corneal injury. Co-culture of rCECs with polysaccharide did not affect cellular morphology or proliferation rate and the application of polysaccharide to the corneal surface *in-vivo* did not stimulate inflammation. These findings are consistent with those of Wu et al who found no adverse effects following injection of the polysaccharide into the subconjunctival space and anterior chamber in rabbits [27].

We found that in vivo application of polysaccharide significantly promoted the recovery of alkali injured corneal epithelium and improved corneal clarity compared to control. Polysaccharide hydrogels are competent to moisture absorption, highly permeable to oxygen, nutrients and other water soluble metabolites [28]. These beneficial features are similar to those with hydrophilic bandage soft contact lenses whose high oxygen permeability is thought to be able to promote epithelial migration and enhance epithelial stromal adhesion [29]. When corneal epithelial cells were co-cultured with polysaccharide, the rate of cell migration was significantly increased, consistent with previous reports [30].

Therapies that rapidly and effectively suppress inflammation and angiogenesis are critical for the treatment of corneal chemical burns [31,32]. In this study we have shown that the application of polysaccharide significantly suppressed inflammation and neovascularization in alkali burn-injured corneal tissue in vivo, consistent with recent reports about inflammation- and neovascularization- suppression activities of polysaccharides [15,16,33,34]. Recent research have attributed corneal epithelium the functions of anti-inflammation and anti-angiogenesis [35], hence the recovery of corneal epithelium promoted by polysaccharide application would further contribute to the suppression of inflammation and neovascularization.

Additionally, our study provided evidence of the efficacy of MSC transplant in the treatment of corneal burns. Subconjunctival injection of MSCs improved the recovery of corneal epithelium, and suppressed inflammation and neovascularization to an extent that was comparable with polysaccharide therapy. These finding are consistent with previous reports that transplanted MSCs had immunoregulatory and anti-angiogenic properties and promoted the healing of corneal chemical burn [18,19,36].

When polysaccharide and MSCs were applied in combination, we found an additive benefit in all parameters examined in this study. Combination therapy resulted in the best corneal re-epithelialization and significantly less corneal opacity at 7 days post-injury compared to monotherapy with either treatment. Combination therapy resulted in sustained suppression of neovascualrisation in contrast to the time-dependent increase observed in the monotherapy and control groups. Corneal bullous keratopath, epithelium distortion, edema, and inflammatory infiltrates were minimal or absent in the combination treatment group in comparison with the more modest benefits observed in the monotherapy groups. These findings indicate that subconjunctival injection of MSCs in combination with topical application of polysaccharide hydrogel is more efficient for the treatment of corneal alkali burns.

With a cutaneous trauma mouse model, Luo et al. found that Hardy Orchid polysaccharide controlled the inflammatory responses and accelerated the wound closure [16]. Diao et al. reported that Hardy Orchid polysaccharide modulated the expression of pro-inflammatory cytokines including NO, TNF-α, and interleukin 1 beta (IL-1β) in a murine macrophage-like cell line [17].

In the current study, application of polysaccharide effectively decreased the expression of key proinflammatory cytokine TNF-α and increased the level of anti-inflammatory cytokine TGF-β, which was consistent with previous report [15]. Furthermore, application of polysaccharide remarkably suppressed the expression of MIP-1α and MCP-1, the major cytokines that regulate migration and infiltration of monocytes/macrophages, indicating that polysaccharide functioned through regulation of critical inflammation related cytokines as well as suppression of leukocytes infiltration.

It was reported that application of MSCs increased IL-10 and TGF-β, and reduced IL-2 and IFN-γ in the damaged cornea, which was accompanied by significantly improved clinical outcomes [36]. MSCs inhibited macrophage infiltration by suppressing the expression of the macrophage chemokine MIP-1α[20] and the proinflammatory cytokine TNF-α production by macrophages [37]. Consistently, our study proved that the application of MSCs increased TGF-β, and reduced the expression of TNF-α, MIP-1α and MCP-1 in alkali injured corneas.

When polysaccharide and MSCs were applied in conjunction, a greater magnitude of change in these parameters was observed, underpinning their additive effect on inflammation suppression.

The preservation of an avascular corneal stroma depends on the balance of pro and anti-angiogenic factors [38–40]. Our study indicates that the ability of polysaccharide and MSCs therapy to inhibit neovascularization is mediated (at least in part) by their suppression of VEGF and MMP-2 expression. As an inhibitor of VEGF and MMP2 expression, TSP-1 was additively up-regulated by polysaccharide and MSCs, which might counteract with VEGF and MMP2 to restore anti-angiogenic balance on the cornea [41,42].

Polysaccharide hydrogels have become attractive focus for clinical applications in recent years due to their efficacy and cost-efficiency in comparison to other therapies. Topical VEGF inhibitors are safe and effective inhibitors of corneal neovascularization [43], however their cost implies financial burden to patients. Therefore, polysaccharide may provide an inexpensive alternative to such treatments. Furthermore, polysaccharide may also serve as a vehicle for drug delivery, facilitating localized administration and greater control of tissue concentrations. Although challenges remain in the clinical application of MSC therapy the field continues to advance. Problems of immunorejection, for example may be addressed by autotransplantation.

In summary, this study provides evidence that Hardy Orchid derived polysaccharide and MSCs are safe and effective treatments for corneal alkali burns and that their benefits are additive when used in combination. Although significant issues may remain in the development of this therapy for clinical application it represents a promising, novel strategy for the treatment of ocular surface injuries.

## Supporting Information

S1 Fig (TIF)

S2 Fig (TIF)

S1 Table (TIF)
