# Supplementary figures and images for: Polysaccharide Hydrogel Combined with Mesenchymal Stem Cells Promotes the Healing of Corneal Alkali Burn in Rats
Source: PLoS One. 2015 Mar 19;10(3):e0119725. doi: 10.1371/journal.pone.0119725 (PMC4366244; doi:10.1371/journal.pone.0119725)

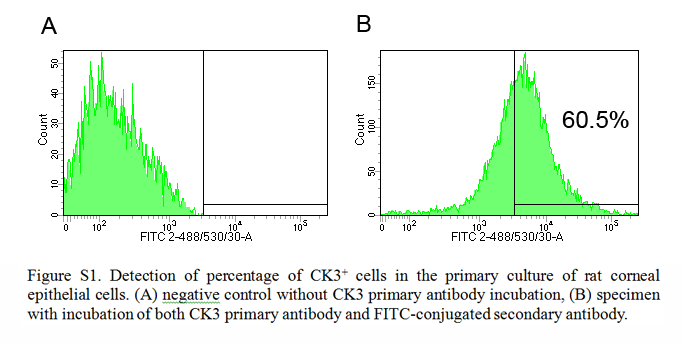

Supplement: S1 Fig — (TIF) [file pone.0119725.s001.tif]

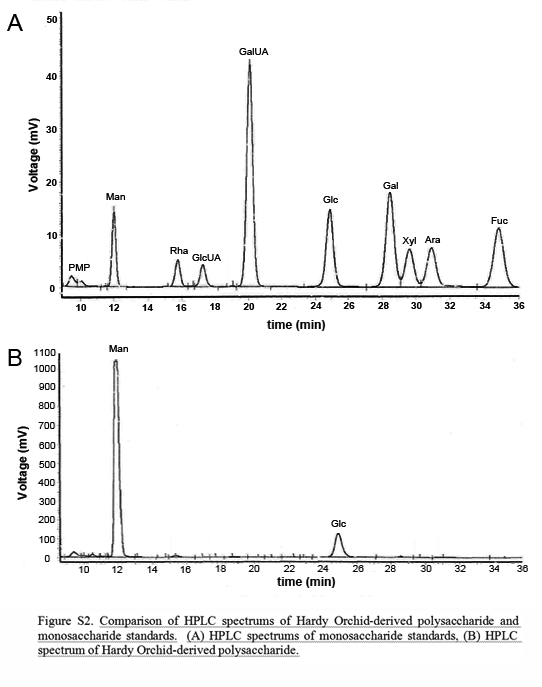

Supplement: S2 Fig — (TIF) [file pone.0119725.s002.tif]

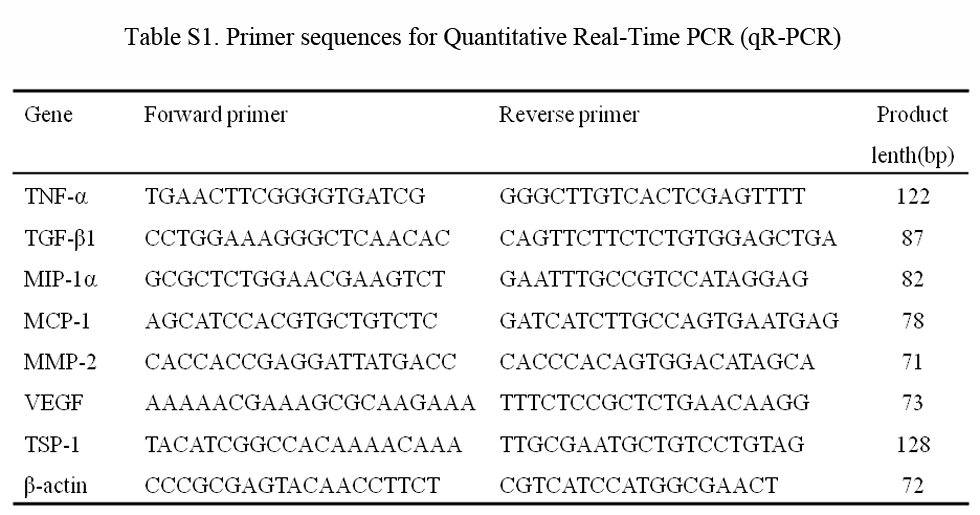

Supplement: S1 Table — (TIF) [file pone.0119725.s003.tif]
